# Correlation between Antistress and Hepatoprotective Effects of Schisandra Lignans Was Related with Its Antioxidative Actions in Liver Cells

**DOI:** 10.1155/2012/161062

**Published:** 2012-06-18

**Authors:** Hao-Jie Pu, Yun-Feng Cao, Rong-Rong He, Zhi-Long Zhao, Jin-Hui Song, Bin Jiang, Ting Huang, Shu-Hong Tang, Jian-Min Lu, Hiroshi Kurihara

**Affiliations:** ^1^Nursing Department, Affiliated Zhongshan Hospital of Dalian University, Dalian 116001, China; ^2^National Population and Family Planning Key Laboratory of Contraceptives Drugs & Devices, Shanghai Institute of Planned Parenthood Research, Shanghai 200032, China; ^3^Pharmacy College, Jinan University, Guangzhou 510632, China; ^4^Department of Surgery, Affiliated Zhongshan Hospital of Dalian University, Dalian 116021, China; ^5^Department of Oncology, The Fifth Hospital of Dalian, Dalian 116021, China

## Abstract

The present study was conducted to investigate the relationship between the anti-stress and hepato-protective effects of Schisandra Lignans Extract (SLE) on stress-induced liver damage. Seven weeks old male mice were fixed in a restraint tube for 18 h to induce liver damage. SLE was orally administered to animals for 5 days at dosages of 100 and 200 mg/kg/day before exposed to restraint stress. Oral administration of SLE significantly reduced restraint-induced liver damage in experimental animal. SLE was further found to significantly alleviate the provocation of corticosterone in stressed mice. SLE also significantly decreased oxidative damage and increased anti-oxidative capability of liver cells by preventing the over production and accumulation of free radicals. In conclusion, the protective effects of SLE on stress-induced liver damage were confirmed, and the correlation between hepatoprotective and anti-stress effects of schisandra lignans was possible related to its alleviation on the malignant effects of stressors for bio-homeostasis, such as balance of oxidation and reduction in cells.

## 1. Introduction

*Fructus schisandrae* (*F. schisandrae*) is the dried mature fruits of *Schisandra chinensis* (Turcz.) Baill. It is regarded as a medical herb in both Traditional Chinese Medicine (TCM) and Western Herbal Medicine (WHM) [1]. In TCM, *F. schisandrae* is used to treat irritability by calming and holding the Qi [2]. Recent pharmacological studies demonstrated that its pharmacological effects in TCM were related with its antistress effects [3]. In WHM, the main application of *F. schisandrae* is in hepatoprotection treatment, used in acute or chronic liver disease and poor liver function [4]. Since the 1970s, the crude herb has been developed as an alternative medicine for the treatment of various liver injuries [5–7]. Chemical investigations indicated the major active compounds were schisandra lignans, including schizandrol A, schizandrol B, and schisantherin A, deoxyschizandrin, schizandrin B, schisandrin C [8], with multiple pharmacological actions [9–12]. However, there are no reports indicated the correlations between the antistress and liver protective effects of *F. schisandrae.*

A serial of studies on the pharmacological actions of schisandra lignans extract (SLE) were conducted recently in our research group. We found that oral administration of SLE significantly reduced stress-evoked anxiety and hepatic metastases in restraint mice [3, 13]. Restraint or immobilization is a common animal model for inducing psychological stress, which results into many nonspecific physiological disorders in autonomic nervous system, endocrine system, and immune system and leads to organ dysfunctions [14, 15]. Our previous studies showed that restraint for 18 h could induce liver damage [16, 17]. Based on these previous studies, the present study was designed to investigate the liver protection effects of SLE on stress-evoked liver damage in mice and explore the possible correlations between the antistress and hepatoprotective effects of SLE.

## 2. Materials and Methods

### 2.1. Preparation of Schisandra Lignans Extract

*F. schisandrae* was supplied by Liaoning Ludan Ltd. (Liaoning, China). A voucher specimen (2009WWZ0006) was deposited in Institute of Traditional Chinese Medicine and Natural Products, Jinan University, Guangzhou, China. SLE was extracted and analyzed by HPLC-MS as previously reported [13]. The compounds of lignans in SLE were identified as schizandrol A, schizandrol B, schisantherin A, deoxyschizandrin, schizandrin B and schisandrin C. Total lignans were quantitated by measuring against schizandrol A standard calibration curve. Each gram of SLE contained 826.3 mg of lignans expressed as Schizandrol A [3].

### 2.2. Animals and Treatments

Seven-week-old male ICR mice were purchased from the Center of Laboratory Animal Science Research of Southern Medical University, Guangzhou, China. All mice were kept in a specific pathogen-free animal room under the controlled condition of temperature 23 ± 1°C with a 12 h light-dark cycle (lights on from 06:00 to 18:00) and were provided with standard laboratory diet and water. The animals were allowed to acclimatize to the environment for 1 week before the experiment. The mice were then divided into normal control group, model control group, and two SLE administration groups. Normal control and model control mice were fed water once daily for 5 days at a dose of 0.1 mL/10 g body weight. SLE was dispersed in water as emulsion of oil before use, and the emulsion was orally administered to animals at 0.1 mL/10 g body weight for 5 days at dosages of 100 and 200 mg/kg/day before exposure to restraint stress. Except for the normal control mice, each mouse was confined to an oval metal restraint tube for 18 h before sacrifice. The care and treatment of the animals conformed to the Guide for the Care and Use of Laboratory Animals published by the US National Institutes of Health, and the experiment was in accordance with animal ethics standards.

### 2.3. Measurement of Plasma ALT Level

Blood and liver samples were collected under ether anaesthesia. Blood samples were centrifuged at 2292 ×g for 10 min at 4°C by a refrigerated centrifuge (Sigma Co., Germany) to obtain the plasma. ALT levels in the plasma were measured by Reitman-Frankel method using a commercial kit.

### 2.4. Plasma Corticosterone Assay by HPLC

Corticosterone is a glucocorticoid produced in response to stress. Plasma corticosterone was extracted from plasma and quantified by HPLC system (Hitachi, Tokyo, Japan). Internal standard of 30 *μ*L cortisone (12.5%, w/v) in methanol-water solution (60 : 40 v/v) was added into plasma (0.7 mL). Steroids were extracted into 2 mL of acetic ether by vortex mixing and immediately centrifuged at 206 × g for 5 min. The organic phase was vortex-mixed with 1 mL of HPLC-grade water. After centrifugation, the organic phase was evaporated with nitrogen at room temperature. The residue was redissolved in 100 *μ*L of methanol-water (60 : 40 v/v). The guard column (5C18, 4.6 × 150 mm; particle size 5 *μ*m; Waters Corp., Milford, Massachusetts) and the column were equilibrated using HPLC-grade acetonitrile-water (38 : 72 v/v) at a flow rate of 1 mL/min. Corticosterone was determined with a UV absorbance detector at 254 nm wavelength.

### 2.5. Measurement of MDA and ORAC Levels in Liver

Liver samples were homogenized in chilled 0.01 M PBS (pH 7.4) using an ULTRA-TURRAX disperser (IKA Co., Germany) and centrifuged at 9,168 ×g for 10 min at 4°C. A 2% liver homogenate was used to determine the protein concentration using a Coomassie brilliant blue kit with bovine serum albumin as the standard. MDA levels in plasma and liver were measured with a commercial MDA kit. A 10% liver homogenate was deproteinized by adding 3% perchloric acid (1 : 1) and centrifuged at 2292 ×g for 15 min at 4°C. The supernatant was kept for further assay. Automated ORAC assay was carried out on a Labsystems Fluoroskan Ascent plate reader (Helsinki, Finland) with fluorescent filters (Infinite F200, excitation wavelength, 485 nm; emission wavelength, 527 nm) as previously described [13].

### 2.6. Measurement of Superoxide Dismutase (SOD) and Glutathione Peroxidase (GPX) Activities in Liver

Total SOD activity was measured by a commercial SOD kit. SOD in samples can inhibit O_2_^•−^ and reduce the level of nitrite. When it reacted with color-developing reagent, the purple-red nitrite can be measured by an MK3 microplate reader (Labsystems Co., Finland) at 550 nm. GPX activity was measured with liver homogenate by a commercial GPX kit. The activity of GPX was calculated by determining the optical density of the enzyme tube and the nonenzyme tube measured by MK3 microplate reader (Labsystems Co., Finland) at 412 nm after GSH had reacted with 5,5-dithiobis(2-nitrobenzoicacid) (DTNB).

### 2.7. Measurement of Expression of CuZnSOD, MnSOD, and GPx mRNA Levels in Liver

Antioxidant enzyme gene expression was semiquantitatively assessed utilizing reverse transcription-polymerase chain reaction (RT-PCR) as previously reported [15]. The PCR primers for mouse CuZnSOD mRNA were (F) 5′-ATGGCGATGAAAGCGGTGTG-3′ and (R) 5′-TTACTGCGCAATC CCAATCAC-3′, and the product size was 456 bp. The PCR primers for mouse MnSOD mRNA were (F) 5′-AAGCACAGCCTCCCAGACCT-3′ and (R) 5′-TCACTTCTTGCAAGCTGTGTATCTT-3′, and the product size was 597 bp. The PCR primers for mouse GPx mRNA were (F) 5′-GAAGTGCGAAGTGAATGG-3′ and (R) 5′-TGGGACAGCAGGGTTT-3′, and the product size was 255 bp. The primers for the mouse housekeeping gene 18 s mRNA were (F) 5′-GGGAGAGCGGGTAAGAGA-3′ and (R) 5′-ACAGGACTAGGCGGAACA-3′, and the product size was 241 bp.

### 2.8. Statistical Analysis

The data were presented as mean ± S.E. Statistical analysis of the data was performed using the SPSS 13.0 statistical package. One-way analysis of variance (ANOVA) was applied to analyze for difference in data of biochemical parameters among the different groups, followed by Dunnett's significant posthoc test for pairwise multiple comparisons. Differences were considered as statistically significant at *P* < 0.05.

## 3. Results

### 3.1. Effects of SLE on Plasma ALT Levels in Restraint-Stressed Mice

**Figure 1:**
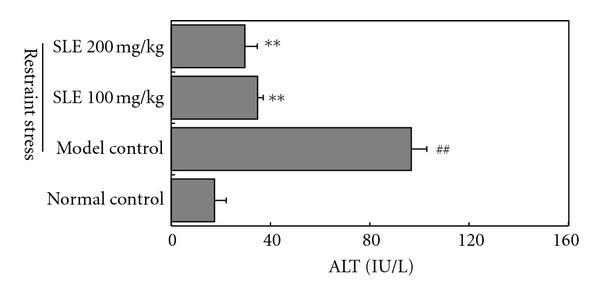
Effects of SLE on ALT levels in plasma obtained from mice loaded with restraint stress. Seven-week-old male ICR mice were fixed in a restraint tube for 18 h before ALT activities assays. The results represent the mean ± S.D of values obtained from 10 animals in each group. The significance of differences from the normal control at ^##^ *P* < 0.01 and model control mice at **P < 0.01.

As shown in Figure 1, plasma ALT level in normal control mice was 17.5 ± 4.7 IU/L, while restraint stress significantly increased the plasma ALT level to 96.7 ± 6.3 IU/L (*P* < 0.01). When SLEs (100 and 200 mg/kg/day) were administered orally to mice for consecutively 5 days before stress, the elevated plasma ALT activity was significantly reduced to 34.76 ± 2.3 and 29.70 ± 5.0 IU/L (*P* < 0.01), respectively.

### 3.2. Effect of SLE on Plasma Corticosterone Level in Restraint-Stressed Mice

**Figure 2:**
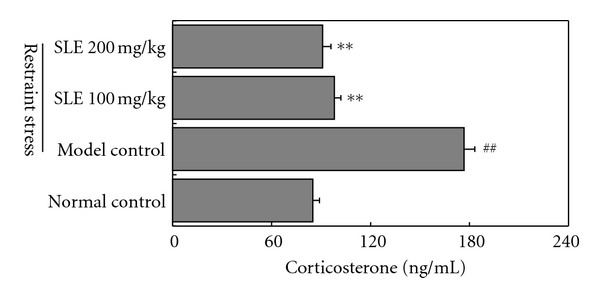
Effects of SLE on corticosterone levels in plasma obtained from mice loaded with restraint stress. The results represent the mean ± S.D of values obtained from 10 animals in each group. The significance of differences from the normal control at ^##^ *P* < 0.01 and model control mice at **P < 0.01.

To determine whether glucocorticoid is involved in restraint-stress-induced liver damage, corticosterone level in plasma was evaluated by the method of HPLC with UV absorbance detector at 254 nm. As shown in Figure 2, plasma corticosterone level in normal control mice was 85.0 ± 4.0 ng/mL, while restraint stress significantly increased the level to 176.7 ± 6.5 ng/mL (*P* < 0.01). When SLEs (100 and 200 mg/kg/day) were administered orally to mice for consecutively 5 days before restraint stress, the plasma corticosterone level was significantly reduced to 98 ± 4.0 and 91 ± 5.0 ng/mL (*P* < 0.01), respectively.

### 3.3. Effects of SLE on Liver Homogenate MDA and ORAC in Restraint-Stressed Mice

**Figure 3:**
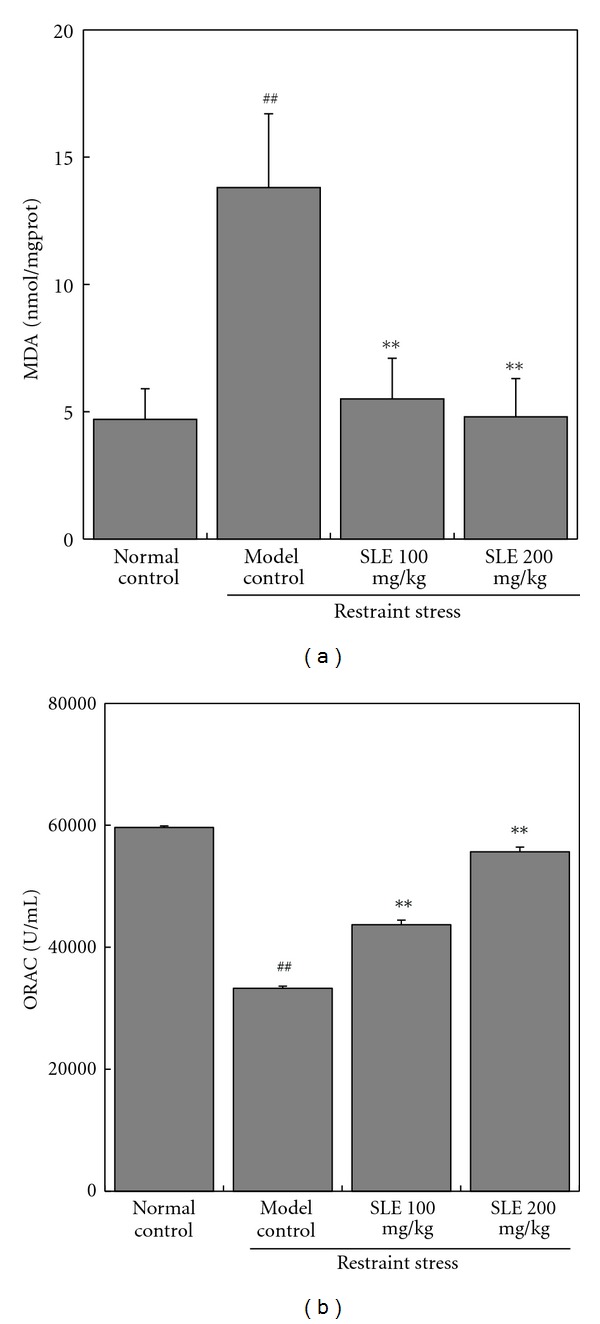
Effects of SLE on MDA (a) and ORAC (b) levels in liver obtained from mice loaded with restraint stress. The results represent the mean ± S.D of values obtained from 10 animals in each group. The significance of differences from the normal control at ^##^ *P* < 0.01 and model control mice at **P < 0.01.

MDA and ORAC of liver homogenate indicated the oxidative stress and antioxidative defense in liver, respectively. As shown in Figure 3, compared to normal control mice, liver damage induced by restraint provoked a significant increment of MDA (from 4.7 ± 1.2 to 13.8 ± 2.9 nmol/mg protein) (*P* < 0.01) and decrease of ORAC level (from 59607.1 ± 253.8 to 33266.8 ± 348.3 U/mL) (*P* < 0.01). However, pretreatment with SLE (100 and 200 mg/kg/day, for 5 days) reduced MDA content to 5.5 ± 1.6 and 4.8 ± 1.5 nmol/mg protein, respectively (*P* < 0.01), and restored ORAC level significantly to 43688.5 ± 750.1 and 55633.0 ± 773.6 U/mL, respectively (*P* < 0.01). Results above indicated that SLE has protective effects on the antioxidative defense of liver in stressed mice.

### 3.4. Effects of SLE on Liver SOD and GPX Activities and mRNA Expressions in Restraint-Stressed Mice

**Figure 4:**
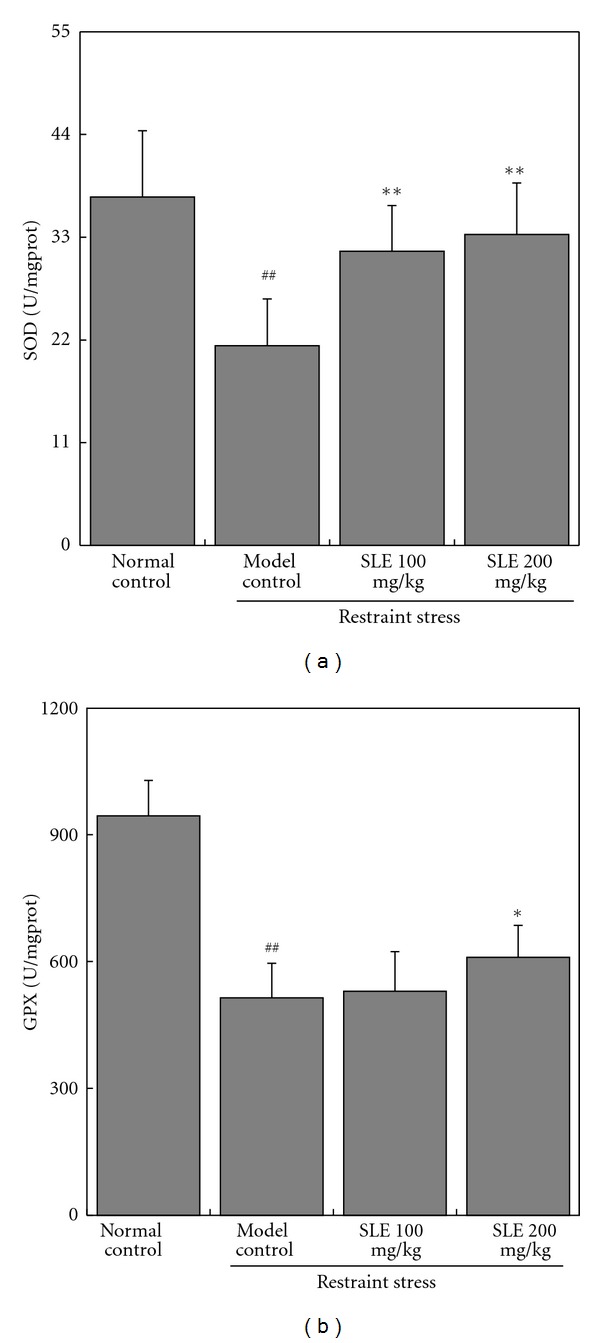
Effects of SLE on liver SOD and GPX activities in liver obtained from mice loaded with restraint stress. The results represent the mean ± S.D of values obtained from 10 animals in each group. The significance of differences from the normal control at ^##^ *P* < 0.01 and model control mice at **P < 0.01.

The activities of important antioxidases were evaluated by determining the SOD and GPx activities. As shown in Figure 4, the basal activity of total SOD activity in liver was 37.3 ± 7.1 U/mg protein. It was markedly decreased to 21.4 ± 5.0 U/mg protein in restraint-stressed mice. Administration of SLE (100 and 200 mg/kg) increased the stress-induced decreases of SOD activity to 31.5 ± 4.9 and 33.3 ± 5.5 U/mg protein, respectively (Figure 4(a)). The GPx activity in liver of restraint-stressed mice was significantly decreased from 944.4 ± 83.7 to 514.3 ± 81.3 U/mg protein. Administration of SLE (200 mg/kg) significantly increased the stress-induced decreases of GPX activity to 609.8 ± 75.8 U/mg protein (Figure 4(b)).

**Figure 5:**
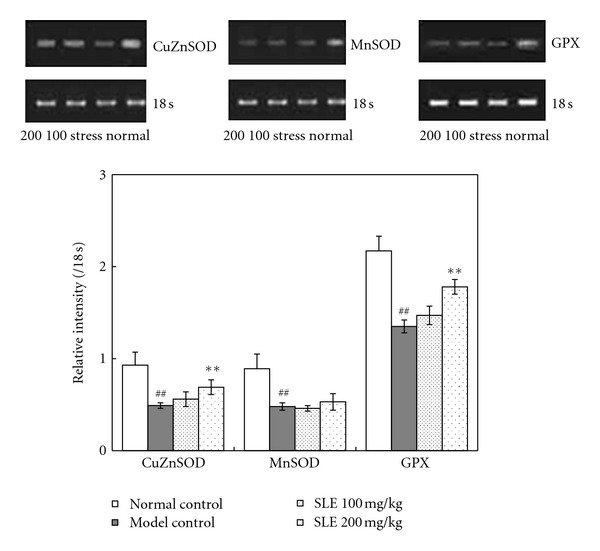
Effects of SLE on SOD and GPX mRNA expression in liver obtained from mice loaded with restraint stress. Densiometric analysis was done on PCR products of SOD1 and SOD2 mRNA expression in mice liver. Results were generated as relative intensity units by densitometry and expressed as the ratio to 18 s. The significance of differences from the normal control at ^##^ *P* < 0.01 and model control mice at **P < 0.01.

The mRNA expressions of antioxidases were further determined by RT-PCR method. The expression of CuZnSOD, MnSOD, and GPx mRNA levels in liver of stressed mice decreased when compared with the control group. Administration of SLE (100 and 200 mg/kg) significantly up-regulated the mRNA levels of CuZnSOD, MnSOD, and GPx (Figure 5).

## 4. Discussion

In the present study, restraint stress caused a significant elevation of plasma ALT in mice, which was consistent with our previous reports [16, 17]. It is known that significantly elevated ALT levels suggest acute liver cell damage, which is caused from the leakage of ALT enzyme into the blood. Our previous studies indicated that mice loaded with restrain for 18 h provoked a mass production of reactive oxygen species (ROS) by inducing an imbalance between the oxidant and antioxidant status. The production of free radicals and peroxides could damage all components of the cell, including proteins, lipids, and DNA [16, 17]. In this study, the significant increase of MDA in liver reflected the oxidative damage of live cell. Although restraint is a well-established psychological stress, the mechanisms of which that caused liver cell damage and production of peroxides were not well defined. Furthermore, the mechanism of protective agents, whether by antistress or free radical scavenging, is still not confirmed.

In this study, when mice were pretreated with 100 and 200 mg/kg/day of SLE for 5 days, the elevated plasma ALT activity was significantly decreased compared with restraint group. This result demonstrated the obvious protection on liver damage induced by restraint. SLE was an active fraction isolated from *F. schisandrae. F. schisandrae* are used to treat acute or chronic liver disease and poor liver function in western herbal medicine [4]. Many modern pharmacological studies proved that *F. schisandrae* could lower the elevated liver transaminases in plasma associated with hepatic dysfunction both in the East and West [7, 18]. In this study, the most abundant ingredients of SLE were schisandra lignans, such as schizandrol A, schizandrol B, schisantherin A, deoxyschizandrin, schizandrin B, and schisandrin C. These isolated lignans from* F. schinensis* have been reported to be capable of lowering elevated transaminases levels in mice induced by CCl4 [19], and the mechanism was proven to prevent the liver from free radicals attack [20]. In the present study, the antioxidative and oxidative extents in liver were determined by measuring ORAC level and MDA contents, respectively. The decrease of ORAC level and increase of MDA contents in liver homogenate suggested that the liver of restrained mice underwent oxidative damage. SLE administration reduced MDA content and restored ORAC level in the liver of restrained mice. These results also implicated that anti-oxidative activity of SLE played an important role in liver protection. However, a recent study investigated the antiradical activities of 14 schisandra lignans using four experimental methods. Unfortunately, these compounds showed weak antiradical activities in concentration range tested [21]. Accordingly, the free radical scavenging activities of schisandra lignans were not enough to clarify its protective effects on liver. However, our results showed that SLE administration played an important role in the activation of antioxidant enzymes, by increasing the activities and mRNA expressions of SOD and GPx in liver of restraint-loaded mice. Therefore, the indirect anti-oxidative activity of SLE by increasing the activities of antioxidant enzymes in liver cell contributed to the protective effects on stress-induced liver damage.

**Figure 6:**
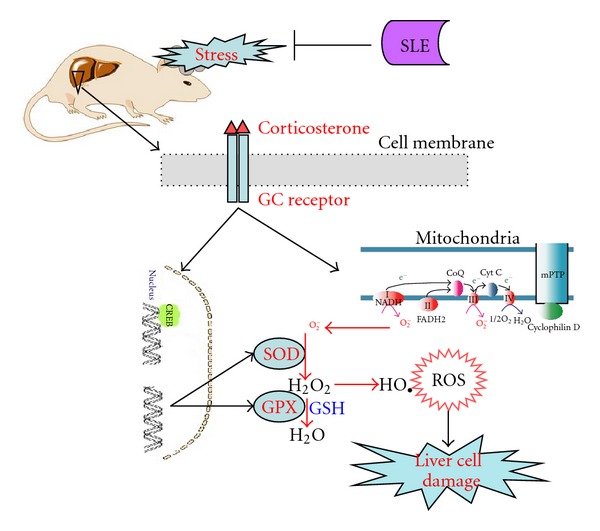
Pathway of SLE effects on stress-induced liver cell damage in mice loaded with restraint. SLE inhibited the provocation of corticosterone in stressed mice. The stress-induced free radicals over production and accumulation were accordingly inhibited.

Apart from activation of ROS scavenger enzymes, SLE also inhibited the activation of ROS production pathways in vivo. In fact, psychological stress that caused oxidative damage was related with provoking of the hypothalamo-pituitary-adrenocortical (HPA) axis. Studies have indicated that upstream of adrenal steroid stress hormones were involved in the downstream of oxidative damage [22]. As shown in Figure 6, restraint stress increased the levels of main glucocorticoid (GC) hormone, corticosterone, in plasma of mice. Many signal pathways, such as PKa, P38MAPK, and NF-*κ*B, were activated when GC hormone was bound with glucocorticoid receptor (GR) [23, 24]. NF-*κ*B regulates several inducible genes, including nitric oxide synthase (iNOS) and other inflammatory cytokines. Evidence has demonstrated that iNOS could produce endogenous nitric oxide (NO) and peroxynitrite (ONOO^−^) [25]. The nitric oxide and peroxynitrite have also been implicated in ROS-mediated damage [26]. The main targets of peroxynitrite are mitochondrial complexes I, II, IV, and V, SOD, GPx, mitochondrial membranes, and mtDNA [27]. Damages of these molecules may induce mitochondrial swelling, followed by permeability transition and in turn damage the liver cell accordingly. In our group, previous reports certificated that restraint stress damaged mitochondrial function, mitochondria membrane potential, and respiratory chain complex activity [16]. For the reasons above, both administration of antioxidants or agents to smooth the stress might protect against restraint stress-induced liver damage. Our results indicated oral administration of SLE significantly resisted the restraint-induced overflow of corticosterone in mice plasma. It was indicated that SLE fought against restrain-provoked activation of stress hormones. A number of studies have demonstrated *F. schisandrae* increased the resistance of laboratory animals subjected to various stress factors using a number of model systems [28, 29]. Our previous study also found that SLE could ameliorate the symptoms of anxiety induced by restraint stress. The mechanisms might be related with its antistress effects via modulating the HPA axis [3]. Therefore, the hepatoprotective effects of SLE may be related to the alleviation of the malignant effects of the stressors.

In conclusion, oral administration of SLE significantly protected mice against restraint-stress-induced liver damage. SLE was further found to significantly alleviate the provocation of corticosterone in stressed mice. In addition, SLE also significantly decreased oxidative damage and increased anti-oxidative capability of liver cells. These results indicated that the protective effects of SLE on liver damage were related to its alleviation on the malignant effects of stressors for bio-homeostasis, such as balance of oxidation and reduction in cells.

## Abbreviations

ANOVA: One-way analysis of variance
DTNB: 5,5-dithiobis (2-nitrobenzoicacid)
GPX: Glutathione peroxidase
HPA: Hypothalamo-pituitary-adrenocortical
HPLC: High-performance liquid chromatography
MDA: Malondialdehyde
ORAC: Oxygen radical absorbance capacity
ROS: Reactive oxygen species
RT-PCR: Reverse transcription-polymerase chain reaction
SLE: Schisandra lignans extract
SOD: Superoxide dismutase
TBARS: Thiobarbituric acid-reactive substances
WHM: Western Herbal Medicine
TCM: Traditional Chinese Medicine.
